# Banana disease-suppressive soil drives *Bacillus* assembled to defense Fusarium wilt of banana

**DOI:** 10.3389/fmicb.2023.1211301

**Published:** 2023-08-03

**Authors:** Huacai Fan, Ping He, Shengtao Xu, Shu Li, Yongfen Wang, Wenlong Zhang, Xundong Li, Hui Shang, Li Zeng, Si-Jun Zheng

**Affiliations:** ^1^Yunnan Key Laboratory of Green and Control of Agricultural Transboundary Pests, Agricultural Environment and Resources Institute, Yunnan Academy of Agricultural Sciences, Kunming, China; ^2^State Key Laboratory for Conservation and Utilization of Bio-Resources in Yunnan, Ministry of Education Key Laboratory of Agriculture Biodiversity for Plant Disease Management, College of Plant Protection, Yunnan Agricultural University, Kunming, China; ^3^Institute of Tropical and Subtropical Industry Crops, Yunnan Academy of Agricultural Sciences, Baoshan, China; ^4^Bioversity International, Kunming, China

**Keywords:** Fusarium wilt of banana, *Foc* TR4, soil microorganism diversity, *Bacillus* spp., biological control

## Abstract

Fusarium wilt of banana (FWB) caused by *Fusarium oxysporum* f. sp. *cubense* tropical race 4 (*Foc* TR4), poses a serious problem for sustainable banana production. Biological control is one of the effective measures to control this destructive disease. High-throughput sequencing of soil microorganisms could significantly improve the efficiency and accuracy of biocontrol strain screening. In this study, the soil microbial diversity of six main banana-producing areas in Yunnan was sequenced by Illumina Miseq platform. The outcome of this study showed the genus of *Chujaibacter*, *Bacillus,* and *Sphingomonas* were significantly enriched in microorganism community composition. Further correlation analysis with soil pathogen (*Foc* TR4) content showed that *Bacillus* was significantly negatively correlated with pathogen content. Therefore, we isolated and identified *Bacillus* from the disease-suppressive soils, and obtained a *B. velezensis* strain YN1910. *In vitro* and pot experiments showed that YN1910 had a significant control effect (78.43–81.76%) on banana Fusarium wilt and had a significant growth promotion effect on banana plants.

## 1. Introduction

Banana is a staple fruit/crop in tropical and subtropical regions of the world, and are planted in more than 130 countries worldwide (D’Hont et al., 2012). China is the second largest producer of banana which is planted mainly in the south and southwest (Li et al., 2019). Yunnan is one of the origin places of banana planting, and banana is also an important economic industry in Yunnan’s tropical and border areas with Laos, Myanmar, and Vietnam (Fan et al., 2021). The banana planting area in Yunnan mainly includes mountain, basin, razed land, river valley, and other landforms, with an average altitude of about 400 m (Yin et al., 2022). The unique ecological conditions make the “highland bananas” popular among consumers (Huang and Xiaojun, 2021).

Fusarium wilt of banana (FWB) caused by *Fusarium oxysporum* f. sp. *cubense* tropical race 4 (*Foc* TR4), elicits a serious problem for sustainable banana production (Butler, 2013). At present, all the banana main producing areas have suffered from this disease in China (Li et al., 2019). The banana industry has been seriously threatened by this pathogen, and it severely restricts banana production in China (He et al., 2021; Li et al., 2021). As a perennial crop irrigated with large amounts of water and fertilizer, the reduction of organic matter and acidification of the soil has gradually become serious, which changed the soil microbial diversity (Wang et al., 2015; Fu et al., 2016; Shen et al., 2019). The community structure composition change of microbes in the soil directly affects the functional diversity and the occurrence and development of soil-borne diseases (Wan et al., 2017; Kwak et al., 2018; Syed Ab Rahman et al., 2018; Carrión et al., 2019). At present, there has been a lot of research on the application of beneficial microorganisms to control the banana fusarium wilt (Damodaran et al., 2020; Jing et al., 2020; Li et al., 2020; Wei et al., 2020). This soil-borne fungal disease could be easily affected by soil microorganisms (Shen et al., 2019). The application of an antagonistic bacteria agent is an effective way to inhibit the occurrence of banana fusarium wilt (Koberl et al., 2017; Wan et al., 2017; Syed Ab Rahman et al., 2018).

The screening methods of beneficial microbes are bent to the direct isolation and culture of microorganisms from soil or plants (Bubici et al., 2019; Chen et al., 2020; Fan et al., 2021). Also, there are other methods such as phospholipid fatty acid (PLFA) map analysis method (Bååth and Anderson, 2003), Biolog ECO-plate detection (Zhang et al., 2009), and others (Bubici et al., 2019), but these methods are time-consuming and easy to obtain miscellaneous microbes. Therefore, the second-generation high-throughput sequencing method can be used to directly detect microbial genes without culture, and obtain detailed classification information of microorganisms, which would greatly improve the screening efficiency of beneficial microorganisms (Huang et al., 2015). There are some in-depth studies in other crops utilizing this method for beneficial microorganism screening, but the application in banana has not successful reports yet.

In this study, six ecological banana plantations representative of the main banana-planting regions in Yunnan Province were selected, based on high-throughput sequencing technology, bulk soil bacteria community structure diversity and difference, real-time fluorescence quantitative PCR, and separation of culturable microorganisms were analyzed of infected/healthy banana bulk soils, verifying the soil biological function of the major bacteria groups through the separation of culturable microorganisms and functional bacteria screening, exploring its biocontrol mechanism of FWB. The screened antagonistic bacterium from bulk soil from this study could provide new approaches for the subsequent ecological control of FWB.

## 2. Materials and methods

### 2.1. Sample collection

Bulk soil samples from 6 banana main producing areas (representative areas of the main banana-planting regions in Yunnan Province, and they have different longitudinal distributions and different climatic conditions.) were collected in Yunnan in 2018, where the disease incidence of FWB was 5–8%. The 5-point sampling method was used to randomly collect bulk soil samples from infected (I) and healthy (H) banana cultivar (Brazilian) in banana fields. Five replicates were collected in each sampling location. The 10 cm-deep soil was collected with a soil collector, and the soil around the roots was collected as the bulk soil. Impurities were removed from the samples such as roots and stones and then sealed with a sterile polyethylene bag and placed in an ice box, transferred to the laboratory to store immediately. The background information of soil samples was shown in Table 1.

**Table 1:** The background information of sampling from different ecological banana plantations.

Sampling sites	Sampling code	Latitude/longitude	Altitude
Yuxi Gejiu Yuanyang Wenshan Dehong Xishuangbanna	YJI1, YJI2,YJI3,YJI4,YJI5 YJH1,YJH2,YJH3,YJH4,YJH5 FKI1,FKI2,FKI3,FKI4,FKI5 FKH1,FKH2,FKH3,FKH4,FKH5 PJI1,PJI2,PJI3,PJI4,PJI5 PJH1,PJH2,PJH3,PJH4,PJH5 LQI1,LQI2,LQI3,LQI4,LQI5 LQH1,BNH2,BNH3,BNH4,BNH5 RLI1,RLI2,RLI3,RLI4,RLI5 RLH1,RLH2,RLH3,RLH4,RLH5 BNI1,BNI2,BNI3,BNI4,BNI5 BNH1,BNH2,BNH3,BNH4,BNH5	23°35′19″N 101°59′29″E 23°12′19″N 102°56′33″E 23°13′25″N 102°45′47″E 22°48′3″N 103°53′13″E, 23°57′33.05″N 97°45′55.31″E, 21°38′6″N 100°43′2″E,	380 m 480 m 480 m 410 m 775 m 620 m

### 2.2. TR4 determination in banana bulk soil by RT-qPCR

TR4 content was determined according to the method described by Bai et al. (2019). Primer FocSc-1 (5′- CAGGGGATGTATGAGGAGGCTAGGCTA-3′)/ FocSc-2 (5′- GTGACAGCGTCGTCTAGTTCCTTGGAG-3′) were used for fluorescence quantitative PCR to detect TR4 content (Lin et al., 2013). The fragment size of the amplification is 242 bp. PCR amplification was performed using Takara SYBR Premix Ex TaqTM (Tli RHaseH Plus) reagent box (Code No. RR820). The standard curve was established by using a recombinant plasmid PMD18T158 containing 158 bp target fragment to determine the concentration of extracted plasmid DNA. The requirements of standard curves were *R*^2^ > 0.99, 90 < Eff% <110.

### 2.3. High-throughput sequencing analysis of soil microorganisms

The total DNA of soil microorganisms was extracted according to the instruction book of Fast DNA SPIN Kit for Soil Kit for soil (MP Biomedicals, United States). The total DNA of the samples was stored at −20°C. Primers 338F (5’-ACTCCTACGGGAGGCAGCAG-3′) and 806R (5’-GGACT ACCAGGGTATCTAAT-3′) were used to amplify the V3-V4 region of bacterial 16S rRNA. The amplified products were sent to Shanghai Personalbio Co., Ltd. and Shanghai Magi Biotechnology Co., Ltd. for high-throughput sequencing on the Illumina Miseq platform.

QIIME (Uparse) 8.0 software was used to process the raw data of Illumina Miseq sequencing. OTUs (Operational taxonomic units) classification was performed at the 97% similarity level. Principal coordinate analysis (PCoA) was conducted according to the Unifrac distance matrix between groups. The heatmap was drawn by the ‘pheatmap package’ of R (V.3.2.5). Multivariate analysis of variance (PERMANOVA) was used to test differences between groups by means of the package (V.2.3–5) in R (V.3.2.5). T-test was used to test the significance between groups, and one-way analysis of variance (ANOVA) was used to obtain the significance of multiple groups. Lefse analysis was performed on differences in bacterial community composition at the phylum and genus levels using Mothur software (*p* < 0.05). R was used to analyze the OTU number of each group, and a Venn diagram was used to show the OTU proportion. The sequence data (accession number PRJNA949429 (16S rRNA)) were downloaded from the NCBI database.

### 2.4. Isolation and antagonist bacteria screening of banana bulk soil

The bacteria isolation was performed according to the following methods: 2 g of soil sample was added into a centrifuge tube filled with 18 mL sterile water. The mixture was oscillated and mixed with a vortex oscillator to obtain the soil suspension, which was then diluted to 10^−7^, 10^−6^, 10^−5^, and 10^−4^ successively by a 10-fold gradient dilution method. One hundred micro liter soil dilutions were inoculated on NA medium (beef extract 3 g, peptone 10 g, agar 15 g, sodium chloride 5 g, for 1 L) for bacteria culture. After 28°C for 24 h culturing, single colonies with different morphology were selected. The bacteria selected were then stored in 50% sterile glycerol at −80°C for later use.

The pathogen *Foc* TR4 (Zhang et al., 2018) was active at PDA medium (potato 200 g, agar 15 ~ 20 g, glucose 20 g for 1 L) and cultured at 28°C for 7 days. Five diameter colony section was transferred to the center of PDA medium with a sterilized punch. The isolated strains were evenly inoculated at four points 25 mm from the center. After 7 days of incubation at 28°C, the inhibitory effect of isolated strains on TR4 was investigated and recorded. PDA plate inoculated only with TR4 was used as the control. The TR4 diameter measurement and inhibition rate were calculated according to Li et al. (2021).

### 2.5. Identification of antagonistic strains

#### 2.5.1. Morphological observation

The isolated strains were cultured on an NA medium and incubated at 30°C for 24 h. Strain characteristics (including morphology, transparency, and color) were carefully recorded, and the micro-morphology character of bacteria was observed under a scanning electron microscope (ZEISS Sigma 300, Germany).

#### 2.5.2. Molecular identification

The isolated strains were inoculated in NB medium and cultured in a shaking incubator at 37°C at 180 rpm for 18 h. Then 1 mL bacterial suspension was centrifuged at 12,000 rpm for 1 min and the bacteria cells were collected. Bacteria DNA was extracted using the Ezup column bacterial genomic DNA Extraction Kit (Sangon Bioengineering Co., Ltd). Primers 27F/1492R (5′-AGAGTTTGATCCTGGCTCAG3′/5′-GGTTACCTTGTTACGACTT-3′) was used to amplify the 16S rDNA gene sequence and Sanger sequencing of bacteria was conducted by Shanghai Personalbio Biotechnology Co, Ltd. The reaction system was as follows: 5 μL 10× buffer (containing 2.5 mM Mg^2+^), 1 μL Taq polymerase (5 u/μL), 1 μL dNTP (10 mM), 39 μL ddH2O, 1 μL DNA template, 1.5 μL upstream primer and downstream primer; Reaction conditions was 95°C, 5 min; 95°C for 30s, 58°C for 30s, 72°C for 1 min 30s, 35 cycles; 72°C for 7 min. The BLAST procedure of the NCBI database was used for sequence alignment analysis, and the phylogenetic tree was constructed using MEGA 7.0 software.

#### 2.5.3. Preparation of biocontrol strain and pathogen fermentation broth

The bacteria were activated at NA medium at 37°C for 24 h. After single colonies were selected and inoculated in NB medium (beef extract 3 g, peptone 10 g, sodium chloride 5 g for 1 liter) at 180 rpm for 48 h at 37°C to obtain fermentation broth. The concentration of strain was diluted by sterile water to 1 × 10^8^ cfu/mL and 1 × 10^7^ cfu/mL, respectively. The activated TR4 strain (Zhang et al., 2018) was inoculated into PDB medium (potato 200 g, glucose 20 g for 1 liter) at 28°C and 150 rpm for 72 h. The suspension was filtered with 4-layer sterile gauze to obtain the spore suspension of TR4 (1 × 10^6^ cfu/mL).

#### 2.5.4. Preparation of pot experiment

The experiment was implemented in the glasshouse at Agricultural Environment and Resources Institute, Yunnan Academy of Agricultural Sciences in April 2021. Banana plants (Brazilian, Cavendish, AAA) grown in quartz stones with 3–4 leaves were transplanted into plastic pots (11 cm × 12 cm) with seedling substrates. Every pot was planted with one banana plant. Until the plant grows to 5–6 leaves, proceed to the next step of the experiment.

#### 2.5.5. Experiment design

Six treatments were set in the pot experiment, which are shown in Table 2. The treatment inoculated with biocontrol strain fermentation broth only was marked as DC1 and DC2, the treatment inoculated with pathogen only was marked as TR4, and the treatment inoculated blank culture medium broth was marked as CK. Among them, treatments I-III were inoculated without TR4, and IV-VI were TR4-inoculation treatments. The pot experiment was set up in three replicates, with 12 plantlets in each treatment.

**Table 2:** The treatments of pot experiment in greenhouse.

Code	Treatments
DC1	Drenched biocontrol strain fermentation broth (1 × 10^8^ cfu/mL) and blank PDB medium
DC2	Drenched biocontrol strain fermentation broth (1 × 10^7^ cfu/mL) and blank PDB medium
CK	Drenched blank NB medium and blank PDB medium
DC1 + TR4	Drenched biocontrol strain fermentation broth (1 × 10^8^ cfu/mL) and TR4 spore suspension
DC2 + TR4	Drenched biocontrol strain fermentation broth (1 × 10^7^ cfu/mL) and TR4 spore suspension
TR4	Drenched blank NB medium and TR4 spore suspension

When the banana plants grew to 6 leaves, 40 mL biocontrol strain fermentation broth (DC1, 1 × 10^8^ cfu/mL) and 10-fold dilution (DC2, 1 × 10^7^ cfu/mL) were drenched on the roots of every potted banana plant, and the control treatment was drenched with 40 mL blank NB culture medium. The spore suspension of TR4 (40 mL, 1 × 10^6^ cfu/mL) was drenched onto the root of banana plants after 7 days of inoculation of biocontrol strain. CK treatment was drenched with a blank PDB culture medium.

#### 2.5.6. Disease and control effect investigation

Disease investigation (leaf and corm symptoms survey) was conducted after 40 days of TR4 inoculation. The disease severity index (DSI) was classified according to Fan et al. (2021). In order to explore the growth promotion effect of biocontrol bacteria, banana plant height, pseudostem thickness, and leaf number were measured 20 days and 40 days after TR4 inoculation referring to the methods of Fan et al. (2021).

### 2.6. Data analysis

SPSS 18.0 software was used for statistical analysis. Duncan’s new complex range method was applied and independent sample T-test were used to analyze the significance of differences among treatments. Means are shown as “Mean ± standard error (S.E.).”

## 3. Results

### 3.1. High-throughput sequencing and OTU cluster analysis

The V3-V4 region of 16S rRNA gene was amplified and sequenced in bulk soil samples of infected (I) and healthy (H) plants from six banana producing areas in Yunnan. The composition ratio of OTUs number of rhizosphere soil samples was shown by a Venn diagram (Supplementary Figure S1). There were 112,365 OTUs in total, 44,077 OTUs were unique in the infected plants’ soil, and 48,859 OTUs were unique in the healthy plants’ soil. Bacterial dilution curves constructed by the number of randomly sampled sequences and the corresponding number of OTUs were flat (Supplementary Figure S2), indicating that bulk soil samples collected from infected and healthy banana plants were reasonably sampled and the bacterial communities’ difference in rhizosphere soil samples could be reflected.

### 3.2. Alpha diversity of soil bacterial communities

The bacterial community of alpha diversity analysis was used to compare the differences of bulk soil bacterial diversity between infected and healthy banana plants in the regional climate types of different producing areas. The results showed that the Ace index and Chao1 index of healthy banana plants were higher than those of infected plants, and the bacterial community diversity in the bulk soil of healthy plants was richer. The bacterial α diversity of infected plants and healthy plants was significantly different (*p* < 0.05). The Shannon index and Simpson index of healthy banana plants were higher than those infested with TR4, but there was no significant difference in the Simpson index (Table 3).

**Table 3:** Diversity of bacterial community in 6 banana producing areas from soil samples derived from Fusarium wilt of banana infected and healthy plants.

Status of plant	Sampling code	OTUs	Chao1	Shannon	Simpson	Coverage (%)
	YJI	1993.42	2069.75	9.87	0.997	0.99
	FKI	3729.78	4143.66	9.73	0.994	0.99
I	PJI	5425.88	5776.17	11.07	0.999	0.98
	LQI	3822.34	4256.56	9.87	0.992	0.99
	RLI	4027.44	4408.29	10.29	0.997	0.99
	BNI	3389.86	3508.97	9.63	0.992	0.99
	Average	3731.45	4027.23	10.08	0.995	0.988
	YJH	1498.2	1562.22	9.03	0.994	0.995
	FKH	4651.9	5149.14	10.58	0.997	0.98
H	PJH	5446.7	6111.04	11.13	0.999	0.98
	LQH	4211.72	4677.63	10.26	0.995	0.99
	RLH	4020.18	4382.60	10.21	0.995	0.99
	BNH	4437.88	4620.12	10.43	0.997	0.99
	Average	4044.43	4417.3	10.27	0.996	0.988

* indicated significant differences in the 0.05 level, ** indicated significant differences in the 0.01 level. YJI indicated the infected soils in Yuanjiang in Yuxi city, FKI indicated the infected soils in Fengkou in Gejiu city, PJI indicated the infected soils in Dapijia in Yuanyang city, LQI indicated the infected soils in Maguan in Wenshan city, RLI indicated the infected soils in Ruili in Dehong city, BNI indicated the infected soils in Xishuangbanna city, Yunnan, China. YJH indicated the healthy (suppressed) soils in Yuanjiang in Yuxi city, FKH indicated the healthy (suppressed) soils in Fengkou in Gejiu city, PJH indicated the healthy (suppressed) soils in Dapijia in Yuanyang city, LQH indicated the healthy (suppressed) soils in Maguan in Wenshan city, RLH indicated the healthy (suppressed) soils in Ruili in Dehong city, BNH indicated the healthy (suppressed) soils in Xishuangbanna city, Yunnan, China.

### 3.3. Bacterial community structure in banana bulk soil

**Figure 1:**
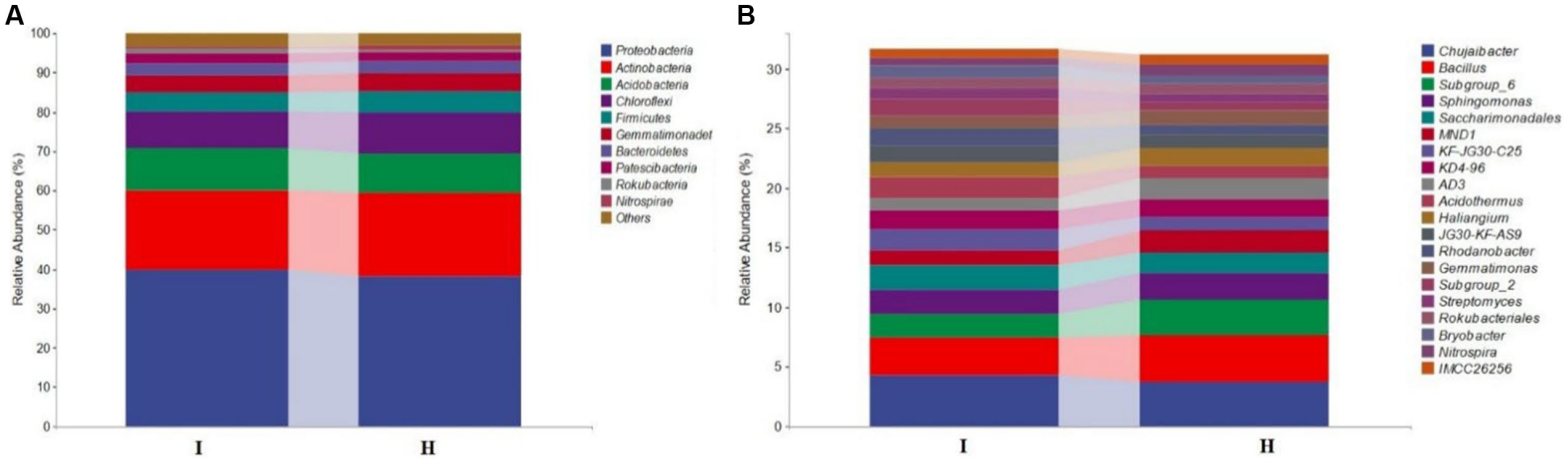
Bacterial community structure of banana bulk soils. Relative abundance of species on phylum level **(A)** and genus level **(B)** of bacteria community in banana infected (I) and healthy (H) soil samples of from 6 banana producing areas.

QIIME software was used to analyze the bacteria community structure of the bulk soil samples from six main banana producing areas. The results showed that there were 10 bacteria species enriched at the phylum level (Figure 1A). Among them, Proteobacteria is the dominant species, accounting for 38.2% (H) and 39.8% (I) of the total number of bacteria. Other dominant bacterial groups were Actinobacteria (21.2% (H), 20.4% (I)), Acidobacteria (10.0% (H), 10.6% (I)), etc. Among the dominant phyla, the abundance of Proteobacteria and Acidobacteria was higher in infected soil (I) than in healthy soil (H), and the abundance of Actinobacteria, Firmicutes, Chloroflexi was higher in healthy soil (H) than in infected soil (I). There were significant differences in bacterial community structure and abundance groups in infected soils (I) and healthy soils (H) at the genus level. The dominant genera were *Chujaibacter* [3.7% (H), 4.2% (I)], *Bacillus* [3.9% (H), 3.2% (I)], *Sphingomonas* [2.3% (H), 2.0% (I)], and *Saccharimonadales* [2.0% (H), 1.7% (I)], etc. (Figure 1B). Among the dominant genera, the abundance of *Bacillus* in healthy soil was significantly higher than that of fusarium wilt-infected soil, which was in accordance with the trend with the Firmicutes.

### 3.4. Cluster analysis of bacterial community composition

**Figure 2:**
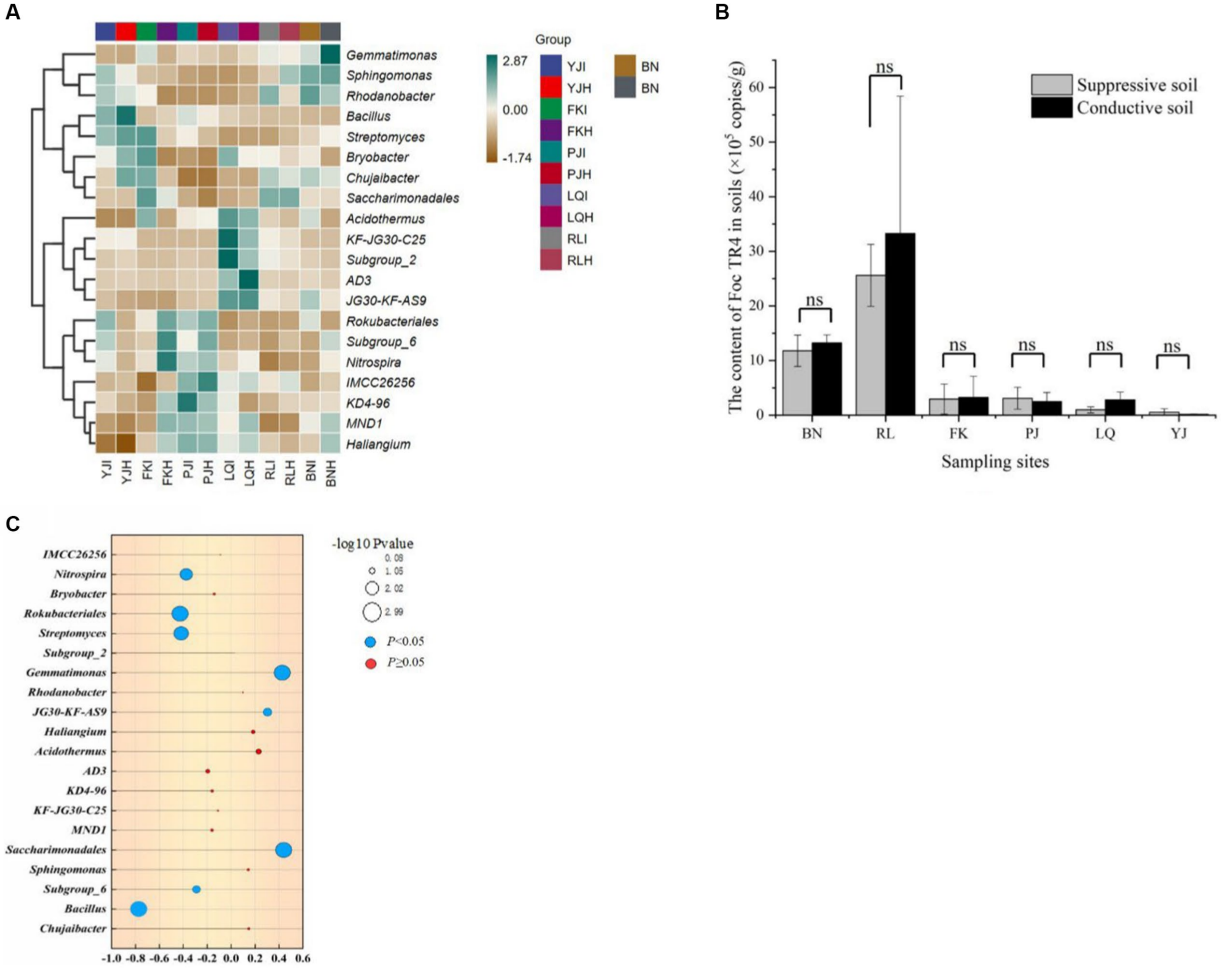
**(A)** Cluster heatmap of main bacteria community in healthy (H) and infected soil (I) samples from 6 banana producing areas. YJ indicated Yuanjiang in Yuxi city, FK indicated Fengkou in Gejiu city, PJ indicated Dapijia in Yuanyang city, LQ indicated Maguan in Wenshan city, RL indicated Ruili in Dehong city, BN indicated Xishuangbanna city, Yunnan, China. **(B)** The content of *Foc* TR4 in healthy (Suppressive) and infected (Conductive) soil samples from 6 banana producing areas. **(C)** Correlation of dominant genus and pathogen content. The X-axis represents the correlation coefficient based on Spearman. The correlation increases with the increase in bubble size. Blue bubbles represent *p* values less than 0.05, Red bubbles represent *p* values greater than 0.05.

At the genus level, hierarchical clustering heatmap analysis was performed using R software for 19 bacterial microbiotas with high relative abundance (> 1%) in bulk soil samples from six main banana producing areas. The bacterial community composition of 60 samples was clustered into two large branches, and 30 samples of infected soil (I) and 30 samples of healthy soil (H) were basically one branch, indicating that their bacterial community composition structure was significantly different (Figure 2A). *Bacillus* was the dominant bacteria in both types of bulk soil and the abundance of *Bacillus* in healthy soil (H) was higher than that of infected soil (I). *Chujaibacter* was also the dominant bacteria in the healthy bulk soil, and *Gemmatimonadaceae* was the dominant bacteria in the infected bulk soil.

### 3.5. Correlation analysis of dominant population microorganisms and pathogen content

Firstly, we detected the content of soil pathogens (TR4) in six main banana producing areas by qPCR. The results showed that the content of TR4 in infected soil was significantly higher than that in healthy soil. Among them, the content of pathogenic bacteria in the Ruili area (RL) was the highest, and the content of TR4 in the Maguan (LQ) and Yuanjiang (YJ) areas was lower (Figure 2B). Correlation analysis was conducted between pathogen TR4 content and soil dominant microorganism’s genus. In the top 20 dominant bacteria genera, the relative abundance of *Saccharimonadales, Gemmatinomas,* and *JG3—KF-AS9* was significantly positively correlated with the TR4 content. *Bacillus*, *Subgroup_6*, *Streptomyces*, *Rokubacteriales,* and *Nietrospira* were negatively correlated with the TR4 content (Figure 2C). We noticed that *Bacillus* not only had significant differences between infected and healthy plants, but also had a significant negative correlation with the pathogen content. Therefore, in order to verify whether *Bacillus* played a key role in the process of induced banana resistance, we carried out the isolation experiment of *Bacillus* genus.

### 3.6. Isolation of *Bacillus* from banana bulk soil

**Figure 3:**
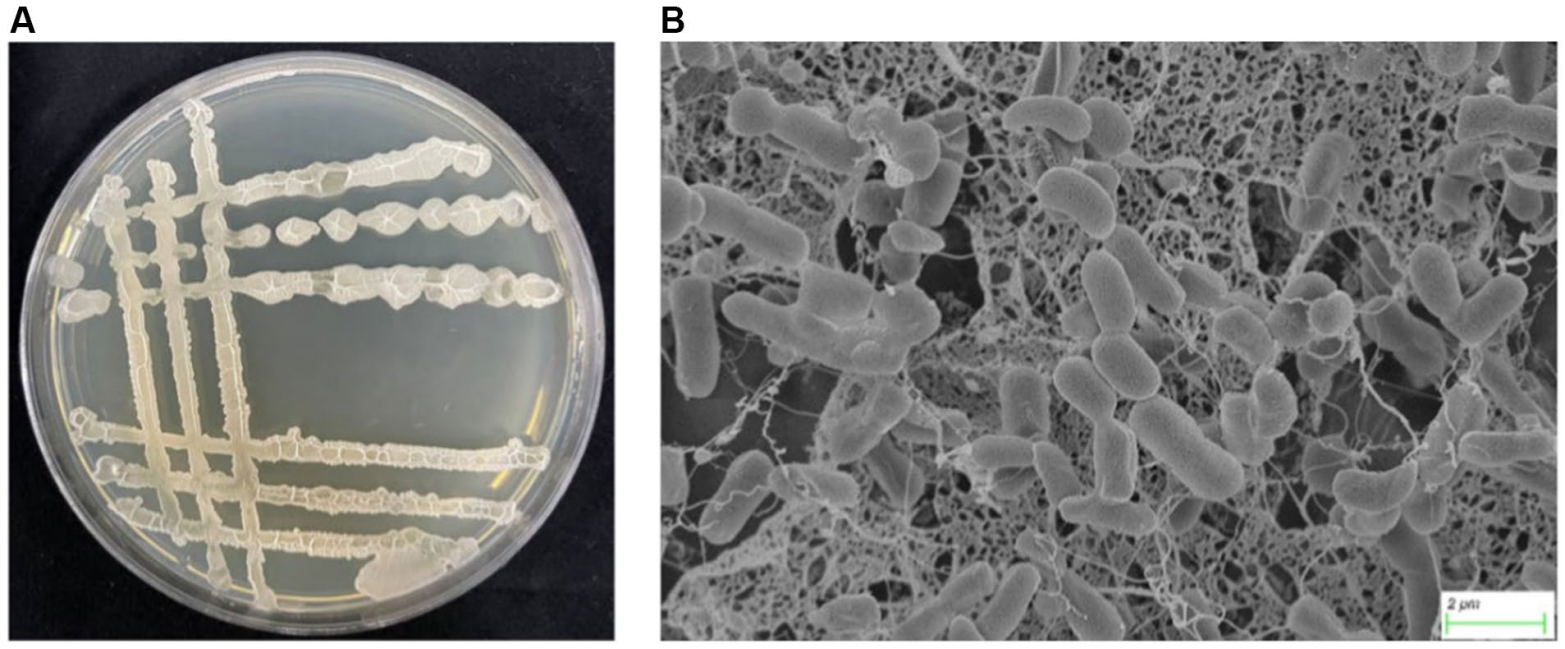
Colony morphology and mycelial morphology of YN1910. **(A)** Colony morphology of YN1910 cultured on NA medium at 30°C for 24 h. **(B)** Scanning electron micrograph of YN1910.

**Figure 4:**
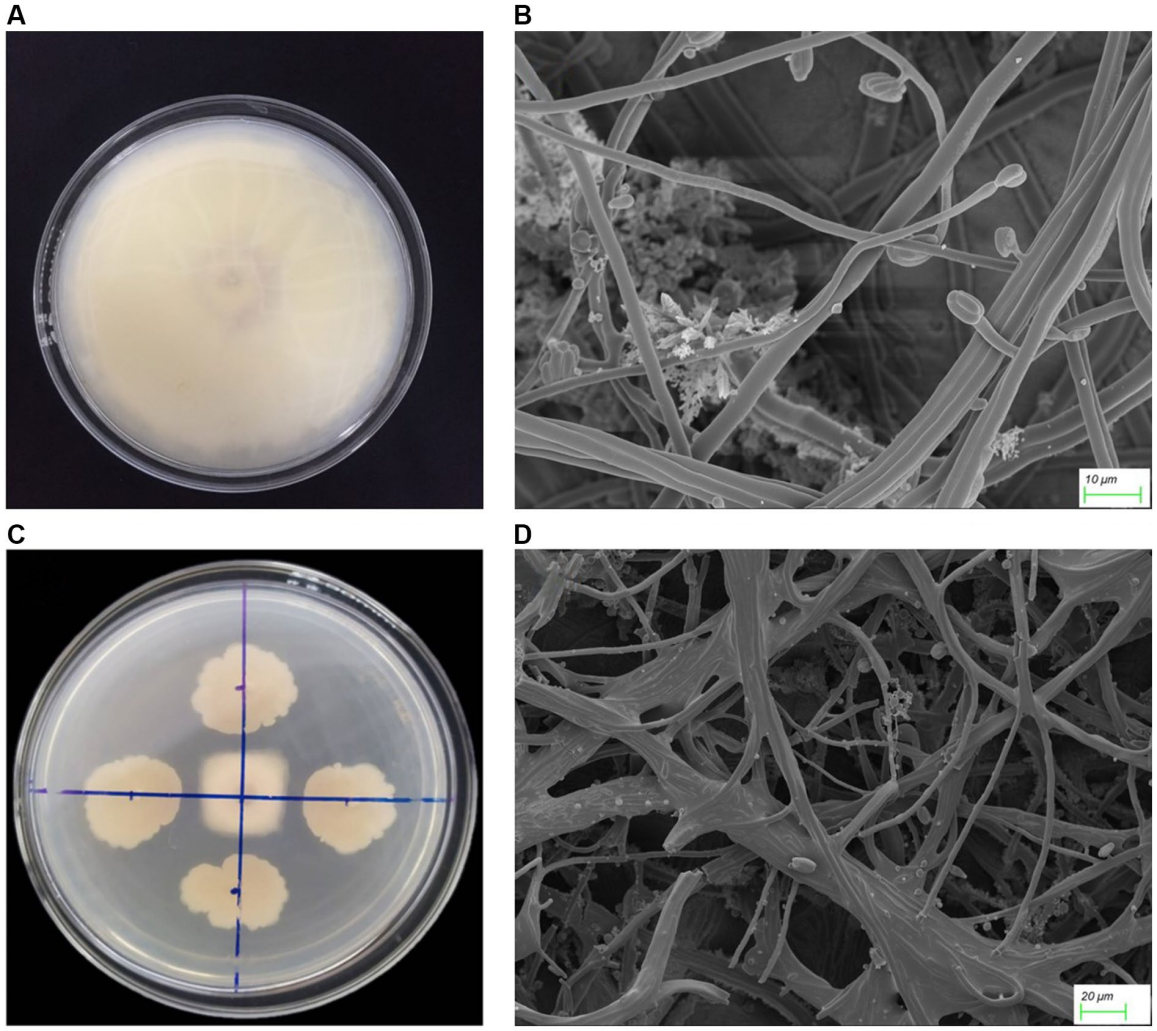
The antagonistic effect of strain YN1910 on TR4. **(A)** TR4 control. **(B)** The antagonistic effect of strain YN1910 on TR4. **(C)** Characteristics of mycelium morphology of control TR4. **(D)** Morphological characteristics of TR4 hyphae after being antagonized by YN1910.

One thousand and one hundred twenty five bacterial strains were isolated from 60 bulk soil in 6 different banana-producing areas in Yunnan. Among these, 594 strains were isolated from the TR4-infected bulk soil, and 531 strains were isolated from healthy soil (Supplementary Table S1). After primary screening, seven *Bacillus* antagonistic strains were screened from infected soil and five *Bacillus* strains were screened from the healthy soil. After dual-culture for secondary screening, one *Bacillus* with the most antagonistic activity against TR4 labeled YN1910 was obtained (Figures 3A,B; Supplementary Table S1). We then observed the colony and microscopic morphology of YN1910. After cultured for 24 h in NA medium, the colony of YN1910 appeared milky-white and convex surface, the edge was irregular and the inside colony was smooth and moist (Figure 3A). Under the scan electron microscopy (SCM), the bacteria looked rod-shaped and rounded-shape at both ends (Figure 3B). To further observe the inhibitory effect of YN1910 on TR4, we conducted the dual-culture for further detection, the results showed that the average diameter of the inhibition zone and inhibition rate of YN1910 against TR4 was 1.63 cm and 81.78%, respectively (Figures 4A,B; Supplementary Table S2). The ‘normal’ TR4 mycelia were smooth and uniform (Figure 4C) under SCM. TR4 mycelium dual-cultured for 7 days culture with YN1910 were also observed under SCM, the results showed that TR4 mycelia were swollen and deformed, the mycelia gradually adhere and begin to dissolve (Figure 4D).

### 3.7. Molecular identification of antagonistic bacteria

**Figure 5:**
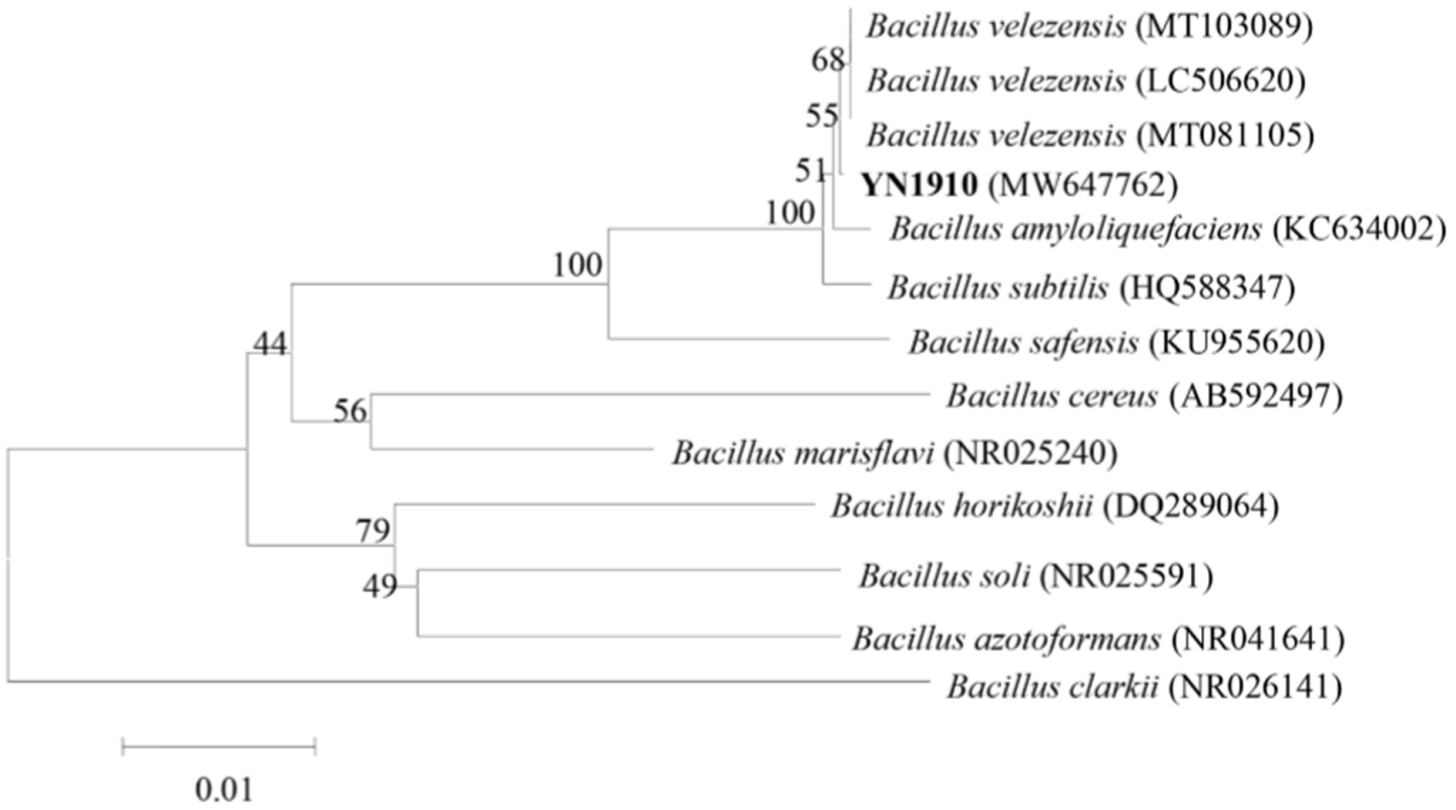
16S rRNA gene phylogenetic tree of YN1910.

In order to determine the species of YN1910 accurately, we further sequenced the 16S region of YN1910. The 16S RNA sequences of strain YN1910 were compared in the GenBank database, and the results showed the highest similarity with *Bacillus velezensis*. Based on a phylogenetic tree of the sequences, YN1910 are related to *B. velezensis* (GenBank Accession No. MW647762) (Figure 5). Through the analysis of the above morphology, and molecular characteristics, the strain YN1910 was identified as a *Bacillus velezensis* (Figures 3–5).

### 3.8. YN1910 had significant control effect on TR4 in greenhouse pot experiment

**Figure 6:**
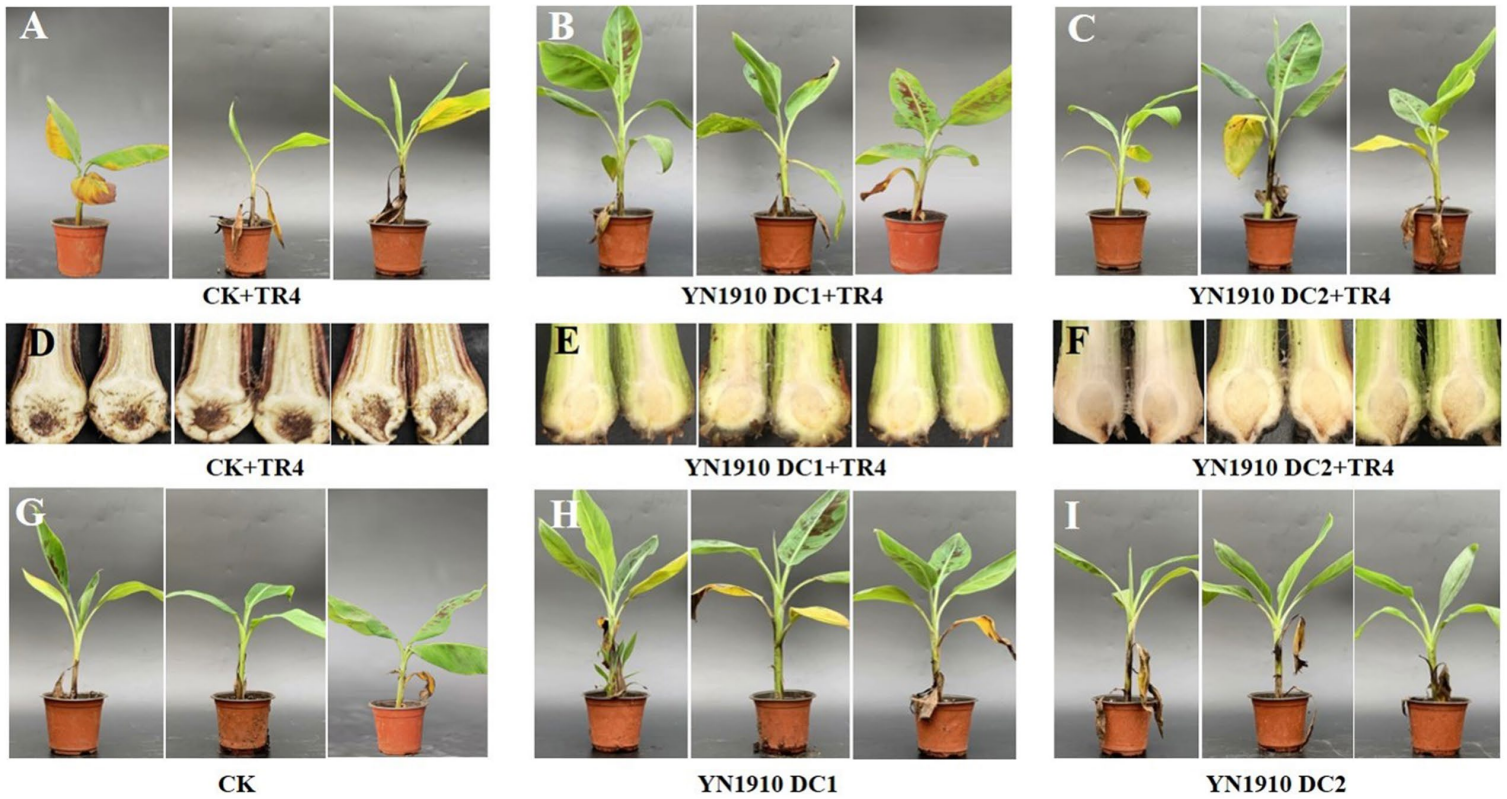
Control effect and growth promoting of antagonistic strain YN1910. **(A)** The banana leaf symptom after being inoculated with TR4. **(B)** The banana leaf symptom after being inoculated with YN1910 (DC1: 1 × 10^8^ cfu/mL) and TR4. **(C)** The banana leaf symptom after being inoculated with YN1910 (DC2: 1 × 10^7^ cfu/mL) and TR4. **(D)** The banana corm symptom after being inoculated with TR4. **(E)** The banana corm symptom after being inoculated with YN1910 (DC1: 1 × 10^8^ cfu/mL) and TR4. **(F)** The banana corm symptom after being inoculated with YN1910 (DC2: 1 × 10^7^ cfu/mL) and TR4. **(G)** The blank control without being inoculated with YN1910 or TR4. **(H)** Growth promoting effect after being inoculated with YN1910 (DC1: 1 × 10^8^ cfu/mL). **(I)** Growth promoting effect after being inoculated with YN1910 (DC2: 1 × 10^7^ cfu/mL).

To further verify the control effect of *B. velezensis* YN1910 on TR4, we conducted pot experiments. Forty days after inoculating TR4, the leaves of the control group (TR4) turned yellow and the banana plants began to wilt (Figure 6A), while the leaves of the treated group (DC1 + TR4, DC2 + TR4) remained green and healthy (Figures 6B,C). After cutting the corms, we observed that the corms in the control group (TR4) became brownish-black in color (Figure 6D), while the treatment corms (DC1 + TR4, DC2 + TR4) were healthy whitish (Figures 6E,F). The disease index on corms of the control group (TR4) was 58.33 ± 4.17, which was significantly higher than that of the treated groups (DC1 + TR4: 10.42 ± 2.08 and DC2 + TR4: 12.5 ± 3.61). The control effects of DC1 + TR4 and DC2 + TR4 were 81.67 and 79.17%, respectively (Supplementary Table S3), indicating that YN1910 had a significant effect on TR4. In addition, we also found that the control effect of DC1 + TR4 was higher than that of DC2 + TR4, indicating that within a certain range, the higher the concentration of biocontrol bacteria inoculated, the stronger the control effect. However, our current evidence indicated insignificant in just two concentration gradients.

### 3.9. Promoting effect of YN1910 on banana growth

Except for the biological control effects on TR4, growth promoting effects were also an important indicator of *Bacillus*. We investigated the growth of banana plants at 0, 20, and 40 days after YN1910 inoculation (Figures 6G–I). The results showed that 20 days after inoculation, the plant height of DC1 (31.74 cm) was significantly higher than DC2 (26.26 cm) and CK (26.95 cm). After being inoculated for 40 days, the plant height of DC1 was 37.15 cm, which was significantly higher than that of DC2 (32.05 cm) and CK (30.33 cm). There was no significant difference of DC2 with CK (Supplementary Figure S3a). As for pseudostem growth, 20 days after inoculation, the pseudostem diameter of DC1 (10.30 mm) was significantly higher than DC2 (8.77 mm) and CK (9.14 mm). When inoculated for 40 days, the pseudostem diameter of DC1 was 11.36 mm, which was significantly higher than that of DC2 (10.26 mm) and CK (9.71 mm). And there was no significant difference between DC2 with CK (Supplementary Figure S3b). Bananas’ leaf growth was also investigated, and the leaf number of DC1 (6.92) was significantly higher than DC2 (6.17) after 20 days of being inoculated YN1910. At 40 days after inoculation, the leaf number of DC1 (7.67) was significantly higher than DC2 (6.83) and CK (6.67), and the leaf number of DC2 and CK had no significant difference (Supplementary Figure S3c). It can be seen that YN1910 could significantly promote the growth of banana plants, and this effect is very significant under high concentrations.

## 4. Discussion

Microorganisms are the key driving force for the formation and transformation of soil contents, the engine of the ecological cycle, which is of great significance to the stability of the entire ecosystem (Veličković and Anderton, 2017; Syed Ab Rahman et al., 2018; Liu et al., 2019). There are many microbial resources with biocontrol potential in the soil, especially in suppressive soil (Xue et al., 2015; Perez-Jaramillo et al., 2016). Although their function is greatly affected by internal and external factors, the use of the characteristics of rapid microbial reproduction, and a large number of artificial reproductions after application into the soil, can regulate the root micro-ecological environment, limit the reproduction of soil-borne pathogens and inhibit the occurrence and development of soil-borne diseases, showing great application potential (Meena et al., 2017).

In the current study, most researchers are bent on the direct screening of antagonistic bacteria and indoor pot experiments for the prevention and control of FWB. Huang et al. (2010) sequenced the FWB-infected soils of banana orchards and normal plantations, they found that the bacterial diversity of soil in normal banana plantations was relatively abundant, among which *Proteobacteria*, *Firmicutes,* and *Acidobacteria* were the main bacterial groups. Deng et al. (2015) compared the diversity of bulk bacterial communities between healthy and diseased banana soils, the results showed that the bacterial diversity of diseased soils was less than that of healthy soils. Shen et al. (2015) and Fu et al. (2012) found that after 2 years of continuous application of microbial organic fertilizer, soil bacterial diversity, and microbial community structure were continuously enriched, thus reducing the incidence of FWB. In this study, *Proteobacteria, Actinobacteria,* and *Acidobacteria* were the dominant group in the infected soil, while *Firmicutes* accounted for more in the healthy soil (Figure 3). Further analysis at the genus level showed that *Bacillus* was the dominant genus in healthy soil that significantly negatively correlated with pathogen content (Figures 4 5). It can be seen that the *Bacillus* in healthy soil is more abundant and which is why it can significantly resist pathogens, but we isolated more *Bacillus* species (seven strains were isolated from infected soil and five strains were isolated healthy soil) from infected soil, which might be due to the higher pathogen content could attracting more antagonistic microorganisms. Therefore, through high-throughput sequencing, we identified the genus *Bacillus* that is most significantly negatively correlated with *Foc* TR4. This has a good indication for the subsequent separation of biocontrol bacteria. According to this idea, we specially isolated and identified antagonistic *Bacillus* genus, and finally obtained a *B. velezensis* strain YN1910 with disease resistance potential in the infected soil of Yuxi.

*B. velezensis* is a heteromorphic species highly homologous to *B. amyloliquefaciens*, which is widely used due to its excellent ability to produce secondary metabolites and colonization in plants (Kang, 2019; Rabbee et al., 2019; Deng et al., 2020). Many strains of this species have been used as pathogen antagonists and plant growth promoters in agricultural production. For example, FZB42, which has high colonization ability in plants such as wheat, tomato, cucumber, and tobacco, has been successfully commercialized for disease control of multiple crops (Chen et al., 2007; Idris et al., 2007; Borriss et al., 2011; Fan et al., 2011; Chowdhury et al., 2013). Many *B. velezensis* have also been shown to have good disease control and growth-promoting effects, like NJN-6, B9601-Y2, CC09 et al. (Yuan et al., 2012; He, 2014; Kang, 2019). In this study, the isolated *B. velezensis* YN1910 was subjected to the controlled greenhouse pot experiment to determine its control effect on FWB. The results showed that YN1910 had a significant mitigation effect on the occurrence of FWB, and the effect was more obvious at high concentrations (Figure 6). This strain also significantly promoted the growth of bananas (Figure 6; Supplementary Figure S1). These results indicated that YN1910 is a successful biocontrol strain, and this is direct information obtained from the results of soil microbial diversity sequencing, which greatly reduces the workload of our isolation of biocontrol strains, and also improves the direction and accuracy of separation.

At present, the biocontrol mechanisms of *B. velezensis* are known through antibiosis, growth promotion, competition for nutrient uptake, and induced host systemic resistance. The biocontrol effects of this type of strain are often the results of the synergistic effect of multiple mechanisms (Bubici et al., 2019). Our research on the prevention and control of FWB by YN1910 is limited to the level of control effect and the specific mechanism is still in progress. And our experiment was carried out in a controlled greenhouse, the application of this biocontrol strain (YN1910) in the field is the next step in our research.

## 5. Conclusion

In this study, the 16S rRNA sequencing of soil microbial diversity showed that *Chujaibacter*, *Bacillus,* and *Sphingomonas* were significantly enriched in microorganism community composition. Correlation analysis with soil pathogen (*Foc* TR4) content showed that *Bacillus* was significantly negatively correlated with pathogen content. So, we isolated and identified a *B. velezensis* strain YN1910 from bulk soil. *In vitro* and pot experiments showed that YN1910 had significant prevention and control effects on FWB. It also had a significant promotion effect on banana growth.

## Data availability statement

The datasets presented in this study can be found in online repositories. The names of the repository/repositories and accession number(s) can be found in the article/Supplementary material.

## Author contributions

HF: conceptualization, designing, performing the experiment, analyzing the data, and writing the paper. PH: performing the experiment, analyzing the data, and preparing the manuscript. SX and SL: performing the pot experiment, and analyzing the data. YW: performing the morphological observation, and analyzing the data. WZ and XL: analyzing the data. HS: performing the physiology experiment. S-JZ and LZ: conceptualization and designing the experiment, reviewing and editing the manuscript, supervising the research, and providing funding support. All authors contributed to the article and approved the submitted version.

## Funding

This study was funded by the earmarked fund for CARS (CARS-31); Yunnan Province Joint Special Project for Agricultural Basic Research (202301BD070001-048); Central government guidance funds for local scientific and technological development (202207AB110009); Yunnan Science and Technology Mission (202204BI090019); Yunling Scholar Programme of Yunnan Provincial Government (YNWR-YLXZ-2018-018); International Atomic Energy Agency under CRP “An Integrative Approach to Enhance Disease Resistance Against Fusarium Wilt (Foc TR4) in Banana – Phase II (D23033)” for Research Contract No. 26673.

## Conflict of interest

The authors declare that the research was conducted in the absence of any commercial or financial relationships that could be construed as a potential conflict of interest.

## Publisher’s note

All claims expressed in this article are solely those of the authors and do not necessarily represent those of their affiliated organizations, or those of the publisher, the editors and the reviewers. Any product that may be evaluated in this article, or claim that may be made by its manufacturer, is not guaranteed or endorsed by the publisher.
