# Supplementary material for: Banana disease-suppressive soil drives Bacillus assembled to defense Fusarium wilt of banana
Source: Front Microbiol. 2023 Aug 3;14:1211301. doi: 10.3389/fmicb.2023.1211301 (PMC10437119; doi:10.3389/fmicb.2023.1211301)
Supplement: Supplementary file 1 [file Table_1.docx]

Table S1. Detailed information of isolated bacteria from bulk soil of six banana planting areas.

| **Collection location** | **Sample type** | **Sample number** | **Total strain number** | **Strain number of primary screening** | **Strain number of secondary screening** |
| --- | --- | --- | --- | --- | --- |
| Yuxi | Bulk soil of healthy banana plants | 5 | 72 | 0 | 0 |
|  | Bulk soil of banana plant infected FWB | 5 | 80 | 2 | 1(YN1910) |
| Gejiu | Bulk soil of healthy banana plants | 5 | 96 | 1 | 0 |
|  | Bulk soil of banana plant infected FWB | 5 | 66 | 1 | 0 |
| Yuanyang | Bulk soil of healthy banana plants | 5 | 87 | 0 | 0 |
|  | Bulk soil of banana plant infected FWB | 5 | 99 | 1 | 0 |
| Wenshan | Bulk soil of healthy banana plants | 5 | 93 | 3 | 0 |
|  | Bulk soil of banana plant infected FWB | 5 | 125 | 1 | 0 |
| Dehong | Bulk soil of healthy banana plants | 5 | 68 | 1 | 0 |
|  | Bulk soil of banana plant infected FWB | 5 | 103 | 0 | 0 |
| Xishuangbanna | Bulk soil of healthy banana plants | 5 | 166 | 3 | 0 |
|  | Bulk soil of banana plant infected FWB | 5 | 150 | 2 | 0 |
| Total |  | 60 | 1205 | 15 | 1 |
