# Supplementary material for: Banana disease-suppressive soil drives Bacillus assembled to defense Fusarium wilt of banana
Source: Front Microbiol. 2023 Aug 3;14:1211301. doi: 10.3389/fmicb.2023.1211301 (PMC10437119; doi:10.3389/fmicb.2023.1211301)
Supplement: Supplementary file 2 [file Table_2.docx]

Table S2. Antagonistic activities of stain YN1910 against TR4.

| Strains | Diameter of TR4 mycelium (cm) | Inhibition rate (%) |
| --- | --- | --- |
| YN1910 | 1.63±0.17 *** | 81.78±0.23 |
| CK(TR4) | 8.97±0.03 | / |
