# Supplementary material for: Banana disease-suppressive soil drives Bacillus assembled to defense Fusarium wilt of banana
Source: Front Microbiol. 2023 Aug 3;14:1211301. doi: 10.3389/fmicb.2023.1211301 (PMC10437119; doi:10.3389/fmicb.2023.1211301)
Supplement: Supplementary file 3 [file Table_3.docx]

Table S3. Control effects of YN1910 on TR4 in the greenhouse pot experiment.

| **Treatment** | **Disease index** | | **Control effect (%)** | |
| --- | --- | --- | --- | --- |
|  | **Corm** | **Leaf** | **Corm** | **Leaf** |
| DC1+TR4 | 10.42±2.08 b | 6.25±3.61 b | 81.67±4.41 | 90.91±5.25 |
| DC2+TR4 | 12.50±3.61 b | 10.42±5.51 b | 79.17±5.07 | 84.24±8.15 |
| TR4 | 58.33±4.17a | 66.67±2.08 a |  |  |
