# Supplementary figures and images for: Banana disease-suppressive soil drives Bacillus assembled to defense Fusarium wilt of banana
Source: Front Microbiol. 2023 Aug 3;14:1211301. doi: 10.3389/fmicb.2023.1211301 (PMC10437119; doi:10.3389/fmicb.2023.1211301)

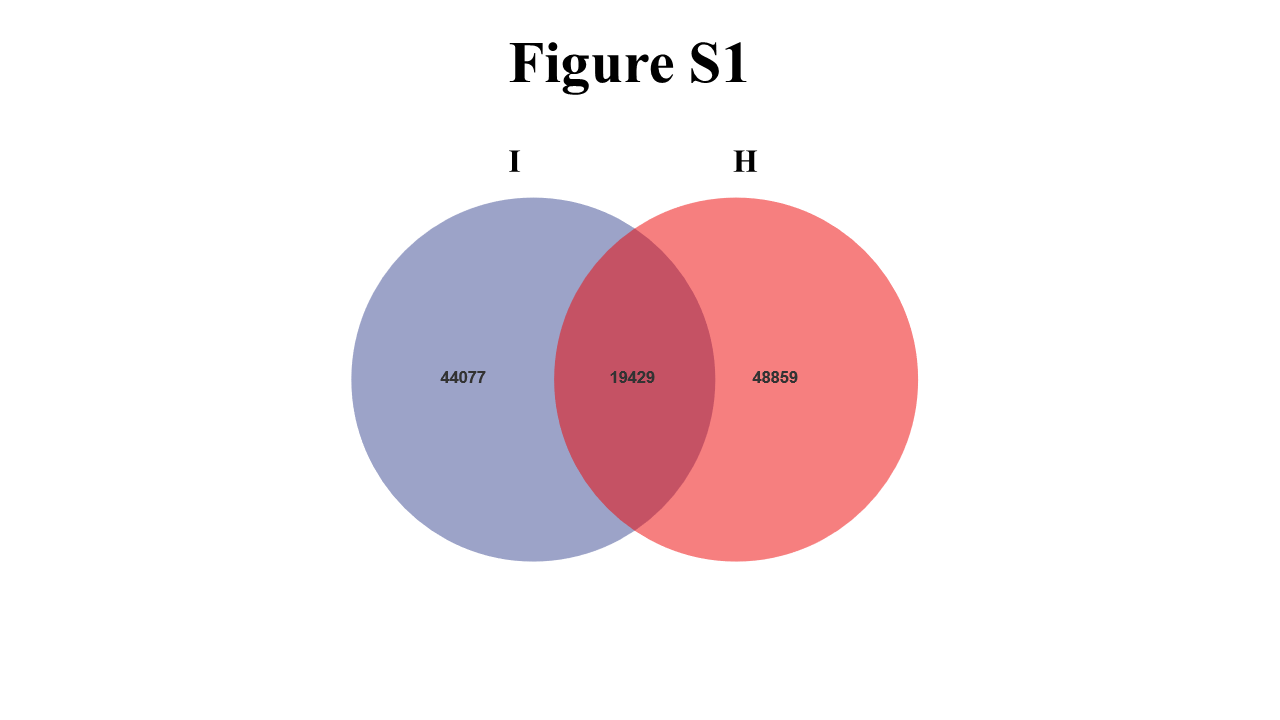

Supplement: Supplementary file 4 [file Image_1.TIF]

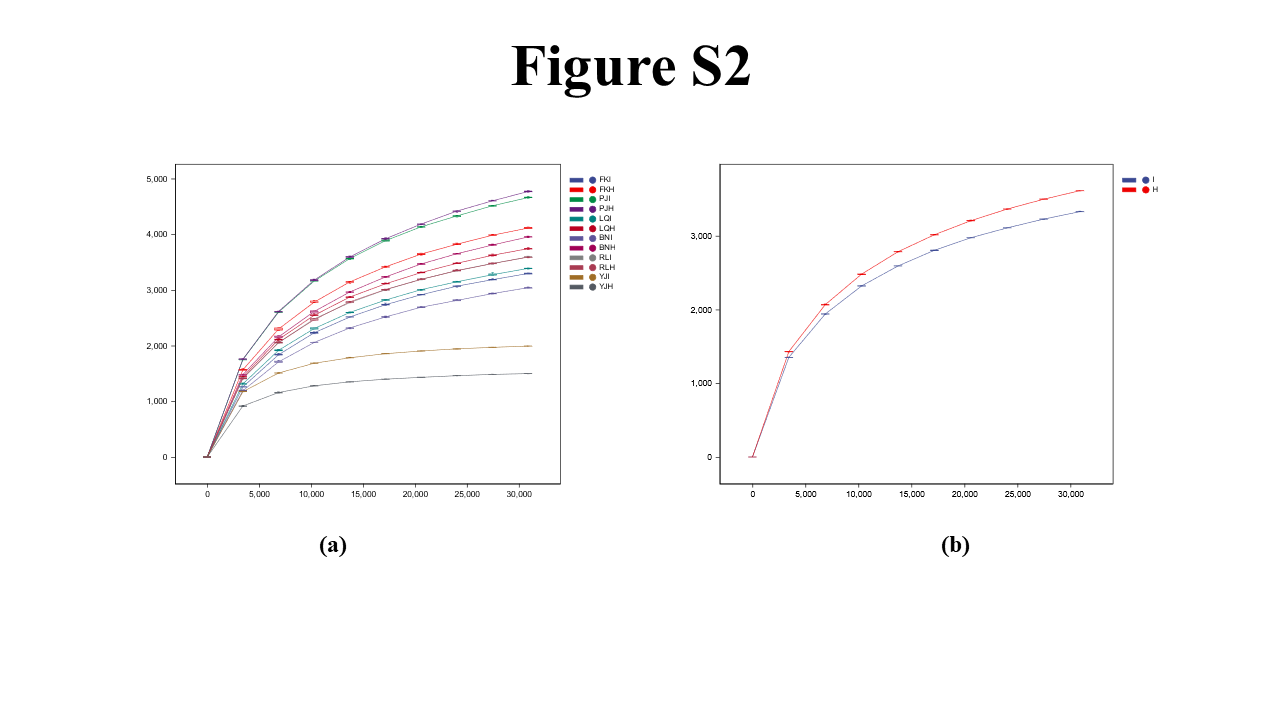

Supplement: Supplementary file 5 [file Image_2.TIF]

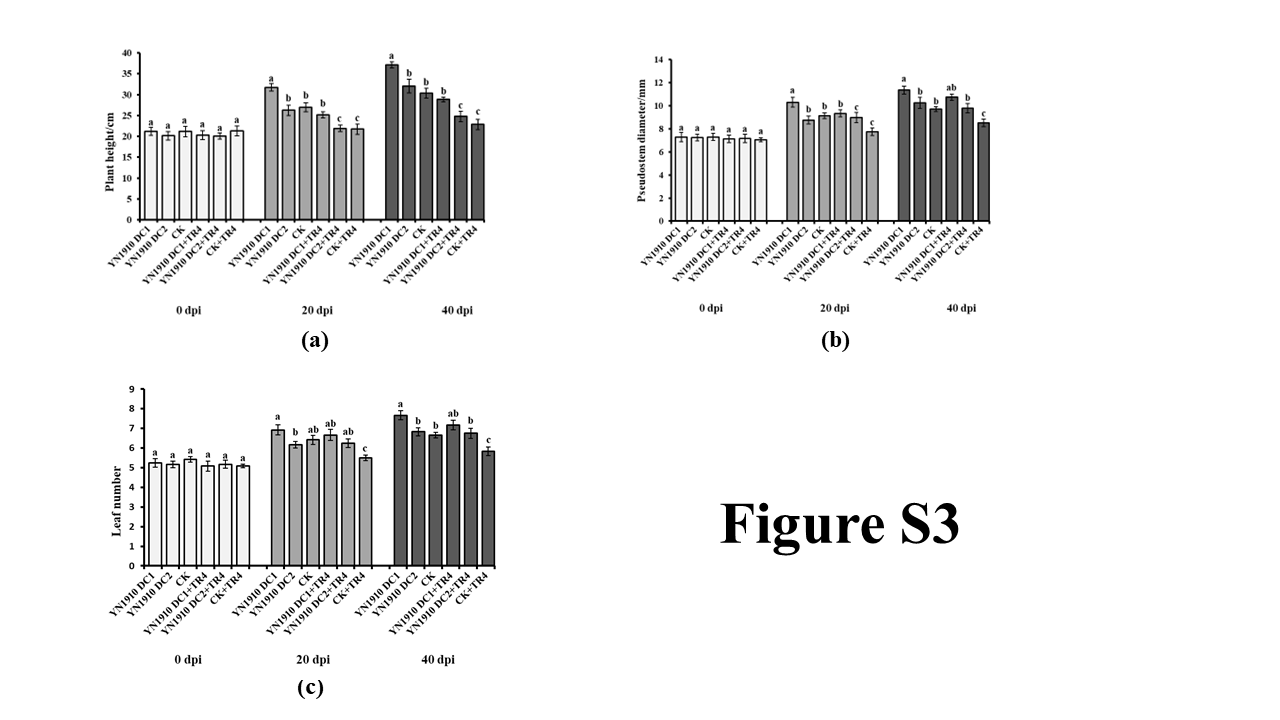

Supplement: Supplementary file 6 [file Image_3.TIF]
